# One Step Forward—The Current Role of Artificial Intelligence in Glioblastoma Imaging

**DOI:** 10.3390/life13071561

**Published:** 2023-07-14

**Authors:** Costin Chirica, Danisia Haba, Elena Cojocaru, Andreea Isabela Mazga, Lucian Eva, Bogdan Ionut Dobrovat, Sabina Ioana Chirica, Ioana Stirban, Andreea Rotundu, Maria Magdalena Leon

**Affiliations:** 1 Doctoral School, Grigore T. Popa University of Medicine and Pharmacy, 16 Universitatii Str., 700115 Iasi, Romania; chiricacostin@gmail.com (C.C.); andreea.rotundu@gmail.com (A.R.); leon_mariamagdalena@yahoo.com (M.M.L.); 2 Department of Oral and Maxillofacial Surgery, Faculty of Dental Medicine, Grigore T. Popa University of Medicine and Pharmacy, 16 Universitatii Str., 700115 Iasi, Romania; 3 Department of Morphofunctional Sciences I, Grigore T. Popa University of Medicine and Pharmacy, 700115 Iasi, Romania; ellacojocaru@yahoo.com; 4 Faculty of General Medicine, Iuliu Hatieganu University of Medicine and Pharmacy, 400012 Cluj-Napoca, Romania; andreeam_isabela25@yahoo.com; 5 Department of Anatomy, Apollonia University, 11 Pacurari Str., 700535 Iasi, Romania; elucian73@yahoo.com; 6 Department of Radiology, Emergency Hospital Professor Doctor Nicolae Oblu, 700309 Iasi, Romania; bogdan.dobrovat@yahoo.com; 7 Faculty of General Medicine, Grigore T. Popa University of Medicine and Pharmacy, 700115 Iasi, Romania; sabinahadimbu@gmail.com; 8 Department of Neurosurgery, Emergency Hospital Professor Doctor Nicolae Oblu, 700309 Iasi, Romania; ioana.stirban@gmail.com

**Keywords:** glioblastoma, brain imaging, artificial intelligence, machine learning, radiomics, deep learning, magnetic resonance imaging

## Abstract

Artificial intelligence (AI) is rapidly integrating into diagnostic methods across many branches of medicine. Significant progress has been made in tumor assessment using AI algorithms, and research is underway on how image manipulation can provide information with diagnostic, prognostic and treatment impacts. Glioblastoma (GB) remains the most common primary malignant brain tumor, with a median survival of 15 months. This paper presents literature data on GB imaging and the contribution of AI to the characterization and tracking of GB, as well as recurrence. Furthermore, from an imaging point of view, the differential diagnosis of these tumors can be problematic. How can an AI algorithm help with differential diagnosis? The integration of clinical, radiomics and molecular markers via AI holds great potential as a tool for enhancing patient outcomes by distinguishing brain tumors from mimicking lesions, classifying and grading tumors, and evaluating them before and after treatment. Additionally, AI can aid in differentiating between tumor recurrence and post-treatment alterations, which can be challenging with conventional imaging methods. Overall, the integration of AI into GB imaging has the potential to significantly improve patient outcomes by enabling more accurate diagnosis, precise treatment planning and better monitoring of treatment response.

## 1. Introduction

Glioblastoma (GB) remains the most common primary malignant brain tumor, accounting for 54% of all gliomas, with a median survival of only 15 months and a 5-year survival rate of 5% [1,2,3,4]. The poor prognosis is due to the high rate of recurrence or tumor progression. The 2021 WHO (World Health Organization) Classification of Tumors of the Central Nervous System has established that GB is a distinct diagnosis from astrocytomas IDH-mutant grade 2, 3 or 4 and must be identified as a diffuse astrocytic tumor in adults that is IDH-wildtype [5,6].

GB is characterized by its extremely invasive nature and rapid progression, leading to poor outcomes for patients. The high recurrence rate also contributes to the increasing mortality of this entity [7,8,9]. Taking this into consideration, early diagnosis plays a crucial role in the therapeutic management of GB. Imaging is an important step in the diagnosis and prognosis of this type of aggressive tumor. Magnetic Resonance Imaging (MRI) can be used as the primary imaging technique. Conventional MRI is the current standard investigation method, particularly contrast-enhanced T1-weighted (CE-T1W), T2-weighted, and fluid-attenuated inversion recovery (FLAIR) images [10]. When it comes to post-operative monitoring, the standard procedure for the determination of tumor growth and response to treatment is through MRI imaging with gadolinium contrast [11]. The excellent soft tissue contrast provided by MRI makes it a suitable imaging technique for distinguishing between various intracranial masses [1]. However, regarding these limitations, the differentiation of multiple kinds of brain tumors through imaging poses difficulties. Precise identification is vital for devising a treatment plan that can enhance the patient’s prognosis, facilitate the grading of tumors, and evaluate their response to therapy [12]. Beyond classical MRI imaging, significant advances have been made in the imaging characterization of GB using artificial intelligence (AI).

**Figure 1:**
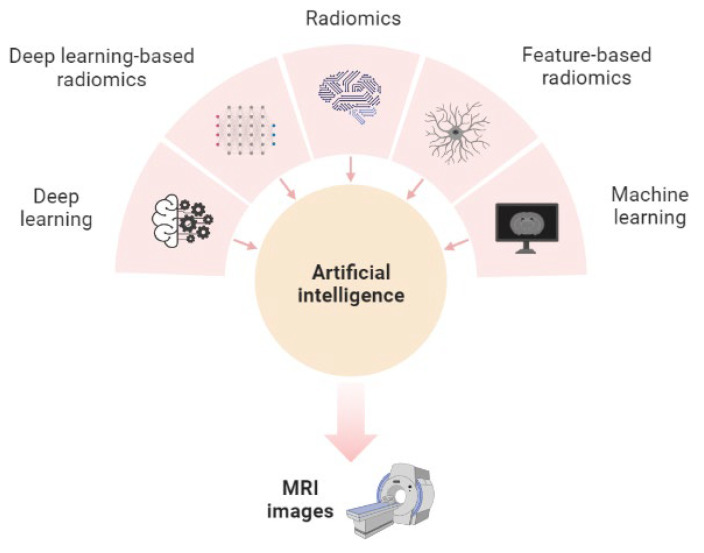
Illustration of potential components of an artificial intelligence model in glioblastoma imaging (created with BioRender.com, accessed on 5 June 2022).

In this regard, AI is the subject of much debate regarding its potential to revolutionize diagnostic imaging. In healthcare, AI is now seen as a new approach to data processing and automated quantitative assessment, with outstanding results in the radiological field. The challenge that remains is represented by the integration of AI algorithms into current hospital practice. Consequently, AI could be used as an important tool in the help of clinicians, raising time efficiency and improving diagnostic precision [13]. Thus, many AI algorithms promise faithful imaging analysis and even increased accuracy compared to classical radiological reports. AI techniques that are becoming more prevalent have demonstrated noteworthy advancements in the realm of medical imaging applications that rely on radiology (Figure 1).

**Figure 2:**
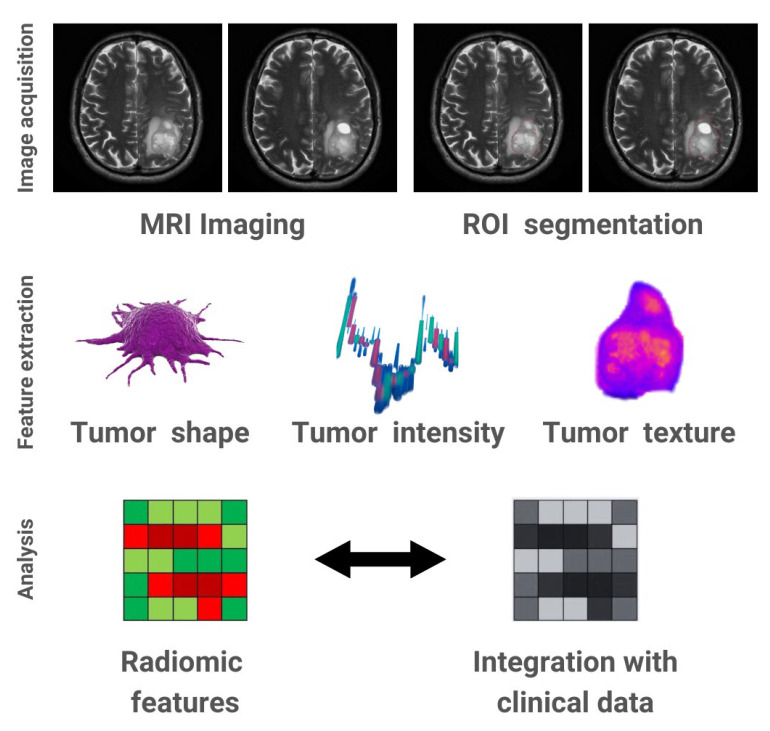
Illustration of the radiomics technique in glioblastoma, including image acquisition, ROI (region-of-interest) segmentation, feature extraction and analysis.

The fundamental principle of AI is relevant to any approach that enables computers to replicate and exceed the boundaries of human intelligence. Automated image processing has become feasible due to advancements in AI technology [12]. Machine learning (ML) and deep learning (DL) are subsets of AI that have the ability to analyze images using different techniques [14,15]. The field of AI has experienced considerable growth in these two domains, complemented by radiomics, a technique that involves extracting a large number of features from medical images to outline tumor characteristics [16]. More specifically, radiomics is a branch of AI that undertakes image manipulation and the extraction of quantitative information related to intensity, shape and texture in order to formulate hypotheses relevant to patient management [3]. In short, the radiomics process involves several steps, starting from image acquisition, segmentation, extraction of features and statistical analysis (Figure 2). However, these steps can differ depending on the research and the purpose [17].

Concretely, the extraction of radiomics features concerns the computation of features as a processing step, where feature descriptors are used in order to assess the characteristics of the grey levels within the region-of-interest (ROI) [18]. There are two distinct subdivisions of radiomics: feature-based and deep learning-based radiomics. Feature-based radiomics involves outlining and separating certain predetermined features from a specific ROI. These features, generally indicated as “handcrafted” or “hand-engineered”, are further selected based on feature-selected algorithms. On the other hand, deep learning-based radiomics requires data generated through learning algorithms for the training of computer models with the purpose of extracting relevant radiomic features [14].

Our paper aims to highlight areas where AI contributes to the imaging diagnosis, prognosis and treatment of GB.

## 2. Materials and Methods

For this narrative review in February 2023, we conducted comprehensive research using the search formula: (glioblastoma OR glioblastoma multiforme OR GBM) AND (artificial intelligence OR AI OR radiomics OR deep learning OR machine learning) AND (magnetic resonance imaging OR MRI OR imaging OR radiology) on Web of Science (1121 results), Scopus (1298 results), and PubMed (935 results) databases. We selected only full-text articles published in the last decade. The most pertinent articles were included in our review after a thorough analysis. During the writing of this narrative review, we carried out additional research to gain a comprehensive understanding of the concepts presented.

## 3. Advances in GB Imaging Using AI

AI has the potential to be a valuable resource that could supplement or even substitute invasive biopsies by utilizing both radiomics and non-radiomics traits in order to contour an accurate classification of brain tumors [1]. Due to recent substantial advances in AI technology, DL algorithms are starting to be increasingly evaluated and used in order to contribute to the diagnostic processes of the medical world. For example, Pasquini and colleagues [19] aimed to build a valid GB-tailored DL model for the prediction of IDH occurrence that allowed for the distinction between IDH-mutant and IDH-wildtype GB using both conventional and advanced MRI data. Discriminating the two types of GB is a critical step because of the discrepancy not only in prevalence (90% IDH-wildtype and 10% IDH-mutant), but also in patient survival (15 months IDH-wildtype and 31 months IDH-mutant), and consequently in patient management. Therefore, finding a non-invasive diagnostic alternative, as opposed to classical invasive biopsy-based methods, has led to increased tendencies toward using AI in neuroradiology. The DL method reached a maximal accuracy of 83% in discriminating the IDH status, proving that radiomics play an ever-increasing role in the future of the clinical environment. Taking all this into account, it is clear that an accurate determination of the tumor type is imperative for subsequent patient treatment [20].

The surgical indication for GB is still a controversial topic; some surgeons recommend only biopsy for diagnostic purposes of the lesion, while others opt for as maximal a resection as possible of the contrast-enhancing formation previously identified on MRI imaging. In a 2020 study, Marcus and collaborators [21] presented an artificial neural network (ANN) that aims to predict a prognosis following surgery, taking into consideration certain anatomical parameters of tumor formation and associated neurological deficit risks. ANN is a type of ML that can be used with small datasets. This study demonstrated that ANN achieved a better prediction of surgical ablation compared to standard systems.

However, GB is a lesion that affects the whole brain, showing infiltrative and diffuse development in the surrounding brain tissue outside the tumor volume itself [22]. Tumor delineation and subsequent surgical resection and radiotherapy planning can be challenging due to the diffuse infiltrative growth pattern of GB, as well as the increased intratumoral heterogeneity that conventional MRI may not adequately capture [2]. While it has been claimed that the combination of AI with multiparametric imaging has the potential to detect tumor cell infiltration in patients with GB, it is better to be cautious about the actual effectiveness of this approach. Although the association of the two methods has been suggested to lead to more precise surgical resection margins and personalized radiotherapy planning, further studies are necessary to determine its true value in clinical settings. Furthermore, it remains to be seen whether this concept can be implemented in a cost-effective and widely accessible manner [2].

In addition, aggressiveness and diffuse infiltrative development are attributes of GB that influence the incidence of early recurrence after standard treatment. Wang et al. [23] developed a preoperative radiomics MRI assessment model in order to evaluate the possibility of early relapse in patients diagnosed with GB. Their retrospective study included a total of 122 patients (65 of whom had an early relapse) randomly divided as follows: 86 subjects in the training cohort and 36 subjects in the validation cohort. Based on clinical features, MRI images, extraction of radiomics information and Visually Accessible Rembrandt Image (VASARI) features of GB, they developed a nomogram. In this manner, the radiomics pattern has been associated with early recurrence in GB patients, and the predictive pattern characterizing the developed nomogram could differentiate patients with early recurrence from patients with later recurrence. Regarding the accomplished results, this model showed that it could be useful in establishing an appropriate treatment course in accordance with the particularities of each case.

Moreover, Shim and collaborators [9] proposed two neural network models to predict local and distant recurrence after maximum surgical resection in patients diagnosed with GB. Based on the inclusion and exclusion criteria, 192 patients were ultimately included in the study. Prediction models were developed based on 32 radiomics features and each patient was stratified into a radiomic risk group. The results showed that the area under the curve (AUC) had a value of 0.969 for local recurrence and 0.864 for distant recurrence for every patient in the validation set. However, this study is retrospective and has a small dataset, which limits its validity. Still, it is a good example of a study protocol that could be improved and developed into prospective multicenter research.

Overall, the literature describes many directions of GB imaging using AI. A significant role in patient management is played by the inclusion of differential diagnoses. However, an exhaustive parallel can often be problematic when only standard imaging is used.

## 4. AI in the Differential Diagnostic of GB

Pathological examination is currently the gold standard for the definitive diagnosis of cerebral lesions, with the disadvantage that it is invasive [24]. Today, the field of AI offers us numerous radiomics, ML and DL models for a non-invasive analysis of brain tumors based on MRI features. These models show promising potential for making an accurate diagnosis and improving the ability to distinguish between various types of cerebral lesions.

The two most prevalent types of brain tumors are GB and brain metastases (BM), each demanding distinctive management. The former is addressed through maximal tumor resection followed by radiotherapy and temozolomide, whereas the latter is commonly treated with stereotactic radiosurgery [1,25]. However, GB and BM share very similar radiological features. As a result, conventional brain MRI is often unable to distinguish between GB and BM, which makes it challenging to differentiate between the two in a clinical setting [7]. Moreover, although advanced MRI characteristics have been increasingly effective in outlining the distinction of GB from BM, no single feature has proven to hold sufficient precision in order to guide clinical decision-making. Several studies have indicated the effectiveness of ML in the extraction of significant radiomics characteristics and classifiers derived from MRI sequences. This allows for an unequivocal differential diagnosis between brain lesions [1]. These results offer valuable diagnostic implications that could be used as reliable assistance in clinical settings. The further management of patients depends highly on the accurate characterization of the detected lesions.

In recent years, numerous studies have focused on the differential diagnosis of GB and BM based on the integration of AI (Table 1).

In addition to BM, other lesions raise difficulties in the imaging differential diagnosis of GB. Even though GB and primary central nervous system lymphoma (PCNSL) are often discriminated against with the help of MRI, these two distinctive neoplasms can sometimes mimic each other. In order to avoid unnecessary invasive interventions and reduce the workload of trained medical professionals, ML is gradually starting to make its way as a very effective tool and is showing great promise in the field of medical imaging. McAvoy and colleagues [31] designed a type of AI called convolutional neural networks (CNN), which has enabled automatic identification of relevant image features in the interest of conducting an accurate (91–92%) classification of these two types of brain tumors, GB and PCNSL, using CE-T1W images. The models developed as a result of their study can be used as an advanced measure, with the help of radiologists in the delivery of precise diagnoses. This is a crucial aspect of reaching the higher objective, that is, the proper and favorable care of the patients.

Furthermore, a retrospective study aimed to integrate an MRI radiomics model into the preoperative non-invasive differentiation of GB from gliosarcoma (GS). This model included radiomics features of both the peritumoral edema and the tumor itself. Although the results of this study were largely favorable, further validation by a prospective study is required. Nevertheless, this approach opens up well-founded, promising perspectives for the differential diagnosis of GB using AI [32]. Another retrospective study [33] aimed to differentiate GB from anaplastic oligodendroglioma (AO) by integrating AI into the diagnostic process. This study included 126 patients, 76 of whom were histopathologically diagnosed with GB and 50 with AO. Based on coherent radiomics information extracted from the MRI images, six diagnostic models were established. The results showed superiority in the diagnosis of GB and AO based on the radiomics methods developed compared to the classical diagnostic approach provided by the radiologist.

Radiomics strategies using ML techniques are not only used in the pursuance of making the distinction between GB and BM, PCNSL, GS or AO, but can also provide the differentiation of GB from radiation necrosis (RN), as a new study intended to prove [34]. Following radiation therapy for a GB, radiation necrosis can develop within 3 years and often looks like a recurrent tumor on an MRI. Consequently, it is crucial to precisely set the two apart, as recurrent GB requires additional anticancer therapies, while RN can be treated conservatively. The objective of this study was to create and validate a radiomics strategy using ML classifiers that perform well, utilizing both conventional imaging and an apparent diffusion coefficient (ADC) map. Despite the similar radiologic appearances of recurrent GB and RN, they each hold unique radiomics characteristics. This information was then extracted and utilized conductive to the development of a clinically relevant predictive model that could differentiate between them. Furthermore, the acquired model could be valuable in determining the appropriate management strategy for patients with the aforementioned conditions after obtaining a clear non-invasive diagnosis.

An extremely important matter is the differentiation of pseudoprogression from early post-treatment tumor progression in GB patients. Along these lines, Kim and colleagues [35] developed a multiparametric radiomics model consisting of radiomics features derived from CE-T1W, FLAIR (fluid-attenuated inversion recovery), ADC, and CBV (cerebral blood volume) data. The model was set side by side with single-layer radiomics models as well as non-radiomics models, ultimately showing significant performance from the multiparametric radiomics model in the diagnosis of tumor pseudoprogression. However, further validation of this model is required, as its accuracy has proven to be moderate (72%). It is certain that such a multiparametric radiomics model promises an accurate diagnosis of tumor pseudoprogression in GB patients with contrast-enhancing lesions during the early post-treatment stage.

On the other hand, the differential diagnosis between tumor progression and pseudoprogression in patients who have undergone total tumor ablation and chemotherapy was suggested to be performed by a non-invasive method based on a neural network in combination with an ML algorithm. Based on MRI imaging (post-contrast T1 sequences) and the acquired clinical data, this proposed ML algorithm shows high accuracy in conducting differential diagnosis in comparison with the efficiency of conventional MRI (using images derived from T1 or T2 sequences) [36]. With the same hypothesis, another study demonstrated greater accuracy resulting from the use of an ML system based on structural features and perfusion sequences as opposed to the classifications performed by radiologists or to those extracted from structural or perfusion MRI imaging analyzed independently [37]. Precise identification of patients presenting with tumor pseudoprogression is essential for their prognosis, as well as for the subsequent clinical decision-making process [38].

There are many other studies in this direction with potentially relevant results for the assertion of an eventual conclusive diagnosis (Table 2).

Indeed, differential diagnosis is crucial not only for diagnostic accuracy, treatment planning, prognosis and management, but also for the prevention of complications and medical errors. AI can be the solution to all of this.

## 5. Discussion

**Figure 3:**
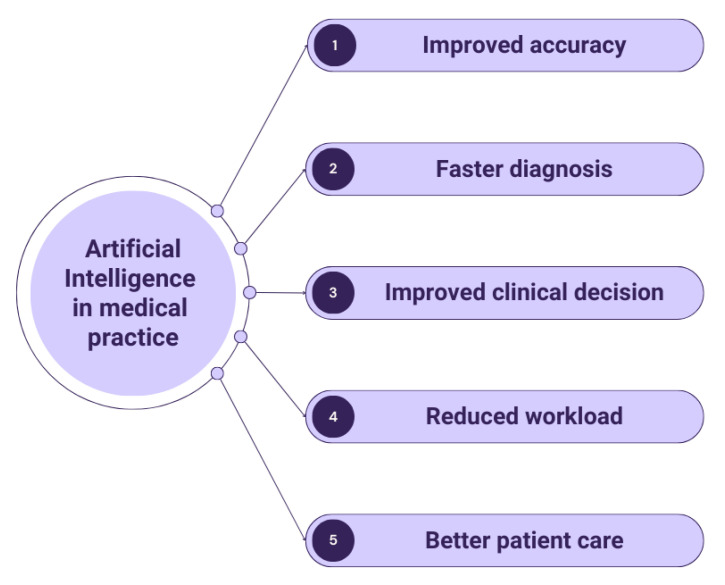
The potential benefits of integrating artificial intelligence into medical practice.

Recently, AI has shown constant anticipative progression. A multitude of artificial intelligence algorithms have already been applied to different aspects of GB imaging in order to integrate the information derived from the gained images with the purpose of developing powerful predictive models. The aim is to incorporate AI into current radiological practice for the final goal of attaining improved diagnosis and consecutive optimal patient management (Figure 3).

In this study, we have presented some of the undeniable directions in which the use of AI holds great potential for GB imaging. AI is a huge step forward in tumor imaging, as well as in non-invasive brain tumor diagnosis, and it proves to be a promising tool in the identification of novel biomarkers and treatment targets. GB imaging integrating AI opens new perspectives on the diagnosis and treatment of aggressive tumors.

We have identified a heightened interest in integrating AI algorithms into the differential diagnosis of GB from BM, with evidently revealing results. For example, Bae et al. [30] found that DL using radiomics features showed the highest performance in distinguishing between the two types of brain tumors. In comparison, the agreement between radiologists, meaning by means of classical radiology reports, was moderate. By making use of information derived from image data that humans alone cannot conduct, ML algorithms can achieve comparable or even better diagnostic performance in contrast to that of clinicians. In this manner, possible human error and bias in medical image interpretation could potentially be overcome. Moreover, Stadlbauer and colleagues [26] self-developed a deep DL technique for differential diagnosis: a one-dimensional CNN, using radiomics features extracted from the cerebral metabolic rate of oxygen as inputs. Their findings performed human interpretation and demonstrated better diagnostic performance compared to the assessments made by radiologists. These results offer valuable diagnostic implications that could be used as reliable assistance in clinical settings, where setting a clear distinction between GB and BM is a crucial aspect in the further management of patients.

Apart from BM, in current medical practice, a greatly important matter is the differentiation of pseudoprogression from early post-treatment tumor progression in GB patients. This is a vital step in the consecutive treatment decision, and AI algorithms could be a key element in this process, exceeding the limitations of human intelligence.

As we have seen, more and more studies are focusing on the fast, accurate and efficient characterization of MRI images illustrating this devastating condition. The results are fascinating and anticipate the prompt inclusion of radiomics and ML models in hospital work.

However, most of the studies published so far are retrospective and have a fairly limited number of participants in the cohorts presented. At the same time, these studies are unicenter, which may be a negative factor in the affirmation of their validity. Thus, no clear conclusions can be drawn about the clinical impact that radiomics and ML models may have. Therefore, these models need further validation through prospective and multicenter studies in order to be approved and applied in current clinical practice.

In addition, most of the proposed AI models are developed from limited data and need to extend the integrated imaging features to be accurately representative on a large scale.

Overall, the current role of AI in GB imaging is rapidly evolving, and with continued advancements in technology and research, it holds great promise for future improvement in the management of GB.

## 6. Future Directions and Conclusions

In the near future, more and more studies will begin to integrate AI in order to demonstrate its validity in medical practice, thus changing the current paradigm in the management of GB and other comparably severe conditions. AI-based models can have a major positive impact on imaging diagnosis and consequent clinical decisions.

With additional research and development, AI has the potential to become a highly reliable instrument for radiologists, neurosurgeons and oncologists, paving the way for greater efficiency and success in the fight against GB.

In conclusion, AI has emerged as a promising tool for improving the accuracy and performance of GB imaging. The use of AI algorithms in medical imaging has enabled the development of automated methods for the detection and characterization of GB, in aid of early diagnosis and treatment planning. Despite its potential, there are still challenges to be addressed, including the need for standardized datasets and the validation of AI models across different patient populations.

## Figures and Tables

**Table 1:** Studies investigating the role of AI in distinguishing between GB and BM.

Study	Purpose	Number of Patients	Training Cohort/ Validation Cohort	MRI Sequences	Features Extracted	Internal/External Validation	Results
Stadlbauer et al., 2022 [26]	The association of radiomics features derived from MR-based oxygen metabolism (oxygen metabolic radiomics) with deep CNN for the differentiation between GB and BM	133	99/34	FLAIR, CE-T1w, DWI, GE-DSC perfusion	74	Internal and External validation	One-dimensional CNN, using radiomics features extracted from the cerebral metabolic rate of oxygen as inputs, outperformed human reading and demonstrated better diagnostic performance compared to the assessments made by radiologists.
Bijari et al., 2022 [27]	The use of a suitable radiomics model to classify GB and BM based on MRI sequences	91	50/41	T1WI, T2WI, CE-T1w, FLAIR	107	Internal validation	Radiomics-based ML can accurately classify GB and BM.
Wu et al., 2022 [28]	The application of DTCWT on routine MRI for the differentiation of GB from BM through the approach of radiomics-based ML	51	27/24	DWI, ADC, FLAIR, T1WI	264	Internal validation	The use of DTCWT has demonstrated the technical prospect of performing feature extraction and dimensional reduction of an image with outstanding results, allowing for the differentiation of GB and BM.
Priya et al., 2021 [29]	Implementation of radiomics-based ML techniques for thedistinction of GB and BM	120	60/60	T1WI, T2WI, FLAIR, DWI, ADC, CE-T1w	1070	Internal validation	For most of the best-performing models across all sequence combinations, the diagnostic performance fell within the range of AUC 0.936 to 0.953.
Bae et al., 2020 [30]	The distinction between GB and BM using a DL–Based model	527	445/82	T1WI, T2WI, CE-T1w, FLAIR	265	Internal and External validation	ML algorithms hold the potential toachieve comparable or even superior diagnostic performance to human readers, findings which could possibly help overcome human error and bias in medical image interpretation.

ADC: apparent diffusion coefficient, BM: brain metastases, CE T1w: contrast-enhanced T1-weighted, CNN: convolutional neural networks, DL: deep learning, DTCWT: dual-tree complex wavelet transform, DWI: diffusion-weighted imaging, FLAIR: fluid attenuated inversion recovery, GB: glioblastoma, GE-DSC: gradient echo dynamic susceptibility contrast, ML: machine learning, MRI: Magnetic Resonance Imaging, T1WI: T1 weighted image, T2WI: T2 weighted image.

**Table 2:** Studies investigating the role of AI in the distinction between pseudoprogression and tumor progression in GB patients.

Study	Purpose	Number of Patients	Training Cohort/ Validation Cohort	MRI Sequences	Features Extracted	Internal/ External Validation	Results
Leone et al., 2023 [39]	The association of conventional MRI, diffusion- and perfusion-derived MRI parameters, as well as radiomic and clinical features using DL in order to differentiate pseudoprogression from progression in GB patients	105	85/20	T1WI, T2WI, CE T1w, FLAIR, DWI, ADC	938	Internal validation	Unsatisfactory results have been reported in terms of the diagnostic performance of the models tested for differentiating pseudoprogression from tumour progression.
Baine et al., 2021 [40]	The application of radiomics techniques with the goal of differentiating pseudoprogression from tumor progression in GB patients on pre-radiation therapy	35	8/27	CE T1w	841	Internal validation	Radiomics shows great potential in predicting the development of tumor pseudoprogression in GB patients using pre-radiation therapy MRI images. Combining radiomic features with clinical factors did not improve predictive performance.
Sun et al., 2021 [41]	The use of an ML strategy in conjunction with radiomics features from CE-T1W imaging for the differentiation of pseudoprogression from tumor progression in GB patients	77	51/26	CE T1w	9675	Internal validation	Radiomics-based CE-T1W imaging demonstrated better results compared to radiologists’ assessment.
Elshafeey et al., 2019 [42]	The use of radiomics features, dynamic susceptibility contrast and dynamic contrast perfusion MRI images for the development of a model that differentiates pseudoprogression from tumor progression in GB patients	98	22/76	T1WI, CE T1w, FLAIR	310	Internal validation	The proposed radiomic model showed satisfactory results with high accuracy, sensitivity and specificity in differentiating between pseudoprogression and tumor progression.

ADC: apparent diffusion coefficient, AI: artificial intelligence, CE-T1W: contrast-enhanced T1-weighted, DL: deep learning, DWI: diffusion-weighted imaging, FLAIR: fluid attenuated inversion recovery, GB: glioblastoma, ML: machine learning, MRI: magnetic resonance imaging, T1WI: T1 weighted image, T2WI: T2 weighted image.

## Data Availability

Not applicable.
